# Far Eastern Scarlet-Like Fever is a Special Clinical and Epidemic Manifestation of *Yersinia pseudotuberculosis* Infection in Russia

**DOI:** 10.3390/pathogens9060436

**Published:** 2020-06-02

**Authors:** Larisa M. Somova, Fedor F. Antonenko, Nelly F. Timchenko, Irina N. Lyapun

**Affiliations:** 1 Somov Research Institute of Epidemiology and Microbiology, Ministry of Science and Higher Education, 690087 Vladivostok, Russia; ntimch@mail.ru (N.F.T.); irina-lyapun@list.ru (I.N.L.); 2 Russian Scientific Center for Roentgen-Radiology, Ministry of Health, 117997 Moscow, Russia; antonenkoff@yandex.ru

**Keywords:** *Yersinia pseudotuberculosis*, infection, epidemic

## Abstract

Pseudotuberculosis in humans until the 1950s was found in different countries of the world as a rare sporadic disease that occurred in the form of acute appendicitis and mesenteric lymphadenitis. In Russia and Japan, the *Yersinia pseudotuberculosis* (*Y. pseudotuberculosis*) infection often causes outbreaks of the disease with serious systemic inflammatory symptoms, and this variant of the disease has been known since 1959 as Far Eastern Scarlet-like Fever (FESLF). Russian researchers have proven that the FESLF pathogen is associated with a concrete clonal line of *Y. pseudotuberculosis,* characterized by a specific plasmid profile (pVM82, pYV 48 MDa), sequence (2ST) and *yadA* gene allele (1st allele). This review summarized the most important achievements in the study of FESLF since its discovery in the Far East. It has been established that the FESLF causative agent is characterized by a unique phenomenon of psychrophilicity, which consists of its ability to reproduce in the environment with its biologically low and variable temperature (4–12 °C), at which the pathogen multiplies and accumulates while maintaining or increasing its virulence, which ensures the emergence and development of the epidemic process. The key genetic and biochemical mechanisms of *Y. pseudotuberculosis* adaptation to changing environmental conditions were characterized, and the morphological manifestations of the adaptive variability of these bacteria in different conditions of their habitat were revealed. The main features of the pathogenesis and morphogenesis of FESLF, including those associated with the *Y. pseudotuberculosis* toxigenicity, were presented. The pathogenetic value of the plasmid PVM82, found only in the FESLF pathogen, was shown.

## 1. Introduction

Enteropathogenic *Yersinia* are known as causative agents of yersiniosis infections (yersinioses) in humans and animals, which are currently attracting attention on a global scale [1]. Pseudotuberculosis in humans until the mid-1950s was encountered as a rare sporadic disease occurring in the form of acute appendicitis and mesenteric lymphadenitis and was known only in European countries and North and South America [2,3]. The detection of the first cases of pseudotuberculosis in people abroad was the result of random findings in lethal septic diseases diagnosed on the basis of a bacteriological study of sectional material. In Europe, the disease usually proceeds as a self-limited acute infection, starting in the intestine and spreading to the mesenteric lymph nodes. *Y. pseudotuberculosis* causes acute enteritis/gastroenteritis, mesenteric lymphadenitis, and diarrheal diseases characterized by fever and abdominal pain, which may resemble acute appendicitis [1,4,5,6]. In Russia and Japan, outbreaks of pseudotuberculosis infection cause serious systemic inflammatory symptoms, and this variant of the disease is called Far Eastern Scarlet-like Fever (FESLF) [7]. Here, *Y. pseudotuberculosis* infection was recognized as a national health problem, which was added to the national notification system in 1988 [8]. Geographical heterogeneity exists between virulence factors produced by European and Far Eastern *Y. pseudotuberculosis* strains. *Y.pseudotuberculosis*-derived mitogen A (YPMa) is a superantigenic toxin produced almost exclusively by Far Eastern strains [9]. Numerous studies have implicated YPMa as a key in the pathogenesis of FESLF.

It is not an exaggeration to say that it was Russia that drew the attention of the whole world to the problem of epidemic pseudotuberculosis, since in 1959 an outbreak of a peculiar infectious disease was registered for the first time in Vladivostok, involving up to several hundred people, originally called Far Eastern Scarlet-like Fever (FESLF) [10]. Only after six years of unsuccessful searches, applying a new method for that time of cold seeding of material from patients, as well as the selfless experience of self-contamination, V.A. Znamensky and A.K. Vishnyakov established that the etiological agent of FESLF is *Yersinia pseudotuberculosis* [11,12]. At the same time, it was stated that this disease, previously unknown in medicine, was a new clinical and epidemic manifestation of pseudotuberculosis in humans, called “epidemic pseudotuberculosis” [11]. For his great contribution to the study of FESLF and prior achievements, in 1989 a group of scientists and specialists led by G.P. Somov were awarded the USSR State Prize. Later, with the introduction of molecular genetic research methods, it was proved that FESLF is associated with a concrete clonal line of *Y. pseudotuberculosis*, widespread in the Russian Federation and characterized by a specific plasmid profile (pVM82, pYV 48 MDa), sequence type (2ST) and *yadA* gene allele (1st allele) [13,14].

In the last 20 years, FESLF has been of interest to researchers around the world due to the fact that this disease belongs to emergent infections at the same time as a particularly dangerous plague infection. As it turned out, the FESLF causative agent, *Y. pseudotuberculosis,* is the genetic progenitor of the plague pathogen *Y. pestis* [15,16], and the transition of *Y. pseudotuberculosis* to *Y. pestis* is accompanied by the loss of many and the acquisition of several genes associated with the pathogenicity of these causative agents. *Y. pseudotuberculosis* was defined as a model system for studying the evolution of pathogens of the genus *Yersinia* and their relationships in the “microorganism-host” system.

This review summarizes the most important achievements of Russian researchers and specialists in the study of Far Eastern Scarlet-like fever over the 60-year period since its discovery in the Far East.

## 2. The Psychrophilicity of *Yersinia pseudotuberculosis* as a Fundamental Property of Sapronoses Causative Agents

*Yersinia pseudotuberculosis* is characterized by a unique phenomenon of psychrophilicity, which consists of its ability to multiply in the environment with its biologically low and variable temperature (4–12 °C), at which the pathogen multiplies and accumulates while maintaining or increasing its virulence, ensuring the emergence and development of the epidemic process [11,17]. The indicated biological feature of this pathogen can explain the confinement of pseudotuberculosis foci to moderate and cold climatic latitudes where the “Far Eastern zone” of yersinioses was distinguished [11,18].

Genetic and biochemical mechanisms of psychrophilicity are carried out at the isoenzyme level, and a low temperature is an inducer that includes the synthesis of “cold” isoenzymes that provide the necessary level of bacterial metabolism with a decrease in the thermal energy of the system [19]. The psychrophilicity of pathogenic bacteria, including *Y. pseudotuberculosis*, has epidemiological and pathogenetic significance [19]. The epidemiological significance of psychrophilicity lies in the fact that it provides the ability of highly virulent bacteria to actively reproduce and accumulate in environmental objects, which qualifies as a reservoir of causative agents of sapronous infections. The pathogenetic significance of the pathogenic bacteria psychrophilicity is that when they live in the environment at a low temperature, they acquire qualities that determine the possibility of the initiation of an infectious process (motility, chemotaxis, adhesion, invasion, resistance to phagocytosis, toxin formation) and contribute to the development of the initial stages of infectious process [20,21,22]. 

The establishment of psychrophilic properties in the FESLF causative agent was the starting point for the study of sapronoses (saprozoonoses), of which *Y. pseudotuberculosis* is a typical representative [23]. As it turned out, the biology of *Y. pseudotuberculosis* bacteria is unique in that they have a dual nature—the ability to both parasitize in humans and animals, and saprophytic existence outside the organism of warm-blooded hosts. Only the continuous circulation of bacteria between them ensures the existence of such pathogens in nature as biological species [24].

The key positions of the modern doctrine of sapronoses [25] indicate that in the process of adaptation to environmental conditions by means of genetic and biochemical mechanisms, an increase in the level of bacteria metabolism in adverse living conditions is provided. The main genetic and biochemical mechanisms of adaptation of sapronoses causative agents to changing ecological conditions include [25]: (1) the ability to reproduce in a wide temperature range (from 0 to 40 °C) due to the synthesis of “cold” and “thermal” isoenzymes that ensure the maintenance of the metabolic level necessary for life bacteria during the transition from environmental objects to the warm-blooded organism and back; (2) the deterioration in the nutritional conditions of model bacteria when excreted from a warm-blooded organism into the environment causes them to rearrange their metabolism from a heterotrophic to an autotrophic pathway, which allows them to assimilate carbon dioxide and other C_1_ compounds and use their carbon to synthesize the necessary cells biopolymers (DNA, RNA, lipids, proteins); (3) with a decrease in the metabolic intensity after the transition of bacteria from the internal environment of a warm-blooded organism to the external environment, the causative agents of sapronoses begin to absorb hydrogen more intensely as a “combustible” material for the respiratory chain of bacterial cells which is associated with the synthesis of adenosine triphosphate, the main energy accumulator in cells.

Thus, the causative agents of sapronoses can multiply and accumulate in environmental objects and, under certain ecological conditions, maintain or increase the virulence of their populations. It was established [25] that conformational changes in the structure of the active surface of enzymes, leading to an increase in catalytic activity which accelerates biochemical reactions in bacterial cells, underlie bacterial adaptation. Under the favorable trophic conditions, the ability of *Y. pseudotuberculosis* to accumulate reserve substances (β-hydroxybutyric acid, polyphosphates) and to consume them under fasting conditions has been proven [26,27].

## 3. Morphological Aspects of the *Y. pseudotuberculosis* Adaptation to Various Living Conditions 

As noted above, adaptive genetic and biochemical mechanisms determine the dual—saprophytic and parasitic—nature of the saprozoonoses causative agents, their ability to reproduce and exist for a long time in objects of soil and water [28,29].

Ultrastructural studies are one of the links confirming the variability of pathogenic bacteria in different living conditions since changes in the physiological state of bacterial cells may reflect changes in their structure. The periodic culture of bacteria is actually a model close to natural conditions since in any phase the bacteria are in a state of restructuring their metabolism in accordance with changing environmental parameters [28]. Until the 2000s, the ultrastructural aspects of the pathogenic bacteria adaptation in the external environment were studied fragmentarily. Then the submicroscopic organization of bacteria was studied exclusively when they were cultured in a thermostat at a temperature of 37 °C, which reflected the state of the pathogen population only under conditions of a warm-blooded organism.

Based on long-term experiments in model microecosystems, we characterized the ultrastructure of the saproonoses causative agents under various trophic and temperature conditions of cultivation [30]. The *Y. pseudotuberculosis* strains of 1 serovar, the main causative agent of FESLF in the Russian Federation, were taken as an object of research. These initial strains, taken from the museum of the Research Institute of Epidemiology and Microbiology Russian Academy of Medical Sciences, Siberian Branch (now: the Somov Research Institute of Epidemiology and Microbiology, Vladivostok), had typical cultural-morphological, biochemical and antigenic properties and were a control during environmental experiments.

To study the influence of biotic and abiotic factors during the habitat of bacteria in the soil, a 1 m^2^ tank was used, filled with soil and located outdoors under natural weather conditions (spring-autumn) [30]. The infectious dose was 10^6^ CFU per 1 g of soil. The effect of temperature on the *Y. pseudotuberculosis* morphology was studied in an experiment using flowing soil columns filled with soil in which inoculated bacteria were kept (infectious dose of 10^9^ microbial cells/mL) at a constant temperature of 6–8 °C and 18–20 °C. To isolate *Y. pseudotuberculosis* bacteria from soil samples, the Serov differential diagnostic medium was used [11]. 

During periodic cultivation of bacteria, a nutrient-rich medium (nutrient broth based on pollock hydrolysate, pH 7.2–7.3) and a medium limited to the main bioelements (0.1 M phosphate-buffered saline) were used at three temperature conditions: 6–8 °C, 18–20 °C and 37 °C. Here, conditions were created that were close to a warm-blooded organism or to the environment [30]. The observation period was 2 years for soil and 40 days for periodic cultures of *Y. pseudotuberculosis*. 

A generalization of the results of studies obtained during model experiments made it possible to establish a complex of ultrastructural changes in the FESLF causative agent under different ecological conditions similar to those in a warm-blooded organism and the environment. It was concluded that these changes in bacteria of periodic and soil cultures that ensure their functional usefulness should be considered as an adaptive response of bacterial populations to changing living conditions, which demonstrates the universal, morphological mechanism of their adaptation [30,31]. A long stay of *Y. pseudotuberculosis* in the soil is provided by phenotypic changes at the ultrastructure level and is expressed in the formation of a capsule and microcapsule, general cover, mucus, intercellular contacts, cytoplasmic outgrowths (prostakes), and accumulation of reserve substances [25,27,32]. Functional adaptation is also provided by an increase in tortuosity and a change in the thickness of the cell wall, variability in the size of bacteria, ribosomal saturation of the cytoplasm and the state of the nucleoid, which reflects conformational changes in bacterial DNA.

Particular attention was paid to the state of the nucleoid. Thus, the bacterial cells of a 7-month-old soil population of *Y. pseudotuberculosis* had a condensed nucleoid consisting of osmiophilic chromatin fibrils. During periodic cultivation of bacteria, chromatin supercoiling was observed in the lag phase and in the stationary growth phase. There is an opinion that such a condensed state of a nucleoid is characteristic of DNA binding with polyamines and has an adaptive value for bacteria [33,34].

It was found that, compared with the control, the electron density of the cytoplasm associated with the number of ribosomes increased in various temperature variants of *Y. pseudotuberculosis* soil cultures, especially in “cold” cultures [30]. An increase in the ribosomal saturation of the cytoplasm of these bacteria was also found in the lag phase of periodic cultivation. A similar phenomenon was associated with a decrease in the productivity of ribosomes, the low efficiency of which was compensated by their additional synthesis to maintain the bacterial growth rate at a certain level. This is consistent with the finding that in *Y. pseudotuberculosis* and some other soil bacteria growing in the cold, the need for nutrients increases and RNA synthesis is enhanced [35].

One of the adaptation mechanisms for nutritional deficiency under suboptimal conditions of existence in bacteria is the synthesis of reserve substances [27,32,36]. In the cultures of *Y. pseudotuberculosis* that we studied, the ability to synthesize polyphosphates and poly-*β*-hydroxybutyric acid (PHBA) was found [30]. During periodic cultivation of *Y. pseudotuberculosis*, polyphosphates were detected in the form of round osmiophilic inclusions, more often along the periphery of a bacterial cell (“necklace”). PHBA inclusions, which were extracted during the alcohol treatment of the preparations, had the form of vacuoles (pseudovacuoles) surrounded by a single-layer membrane. They were found in periodic cultures of *Y. pseudotuberculosis*, regardless of the composition of the medium and growth phase, but more often in bacteria that lived in the soil at all observation times.

Finally, during periodic cultivation, structures were discovered that were not inherent in bacteria when they lived in the soil. Under the conditions of the “cold” cultivation of *Y. pseudotuberculosis,* a complex of common features was found: electron-dense deposits on the cell wall and electron-dense “flocculent” masses in the nucleoid zone and in the cytoplasm [30]. Based on an analysis of own and published data [37], we suggested that these ultrastructural changes reflect the metabolic activity of bacteria associated with their production of biologically active substances under conditions of functional adaptation.

The presented materials demonstrate the morphological basis for the adaptation of sapronoses causative agents to different living conditions corresponding to the saprophytic and parasitic phases of their existence.

## 4. The Main Features of Pathogenesis and Morphogenesis of FESLF

Far Eastern scarlet-like fever is an acute generalized infectious disease characterized by a cyclic course, severe toxic-allergic syndrome, and predominant damage to the gastrointestinal tract, liver and joints [10].

Systematization of literature data on the development of the infectious process during pseudotuberculosis infection, as well as the results of our experimental studies on a nosological, oral infection model caused by *Y. pseudotuberculosis* strains isolated from patients with FESLF, allowed us to substantiate the modern concept of the patho- and morphogenesis of this disease [38,39]. The following phases of the FESLF pathogenesis were distinguished: (1) the phase of contaminating the mucous membranes of the digestive tract; (2) the reproduction phase of the pathogen in the entrance gate with the development of primary focal changes (pharyngitis, tonsillitis, gastroenteritis, enteritis); (3) the phase of primary bacteremia, hematogenous and lymphogenous dissemination of the pathogen; (4) the phase of secondary focal changes (terminal ileitis, appendicitis, ileotiflitis, hepatitis, etc.) and allergization of the organism; (5) a phase of repeated bacteremia with relapses and exacerbations of infection; (6) the phase of sanitation of the organism and repair. With septic complications, lethal outcomes occur.

Thorough bacteriological studies have shown [23] that at the earliest stages of the infectious process (within the first hour after oral infection of experimental animals), *Y. pseudotuberculosis* penetrated the circulatory system, causing primary bacteremia and hematogenous dissemination of the pathogen.

The most justified selection of the following clinical-morphological forms of FESLF are [39]: (1) abdominal forms—gastroenteritis, enteritis, mesadenitis, appendicitis, hepatitis, pancreatitis; (2) extra-abdominal forms—pneumonia, myocarditis, meningoencephalitis, nephritis; (3) exanthematous forms—scarlet-like form and nodular erythema; (4) arthralgic form; (5) combined form; (6) septical form; (7) erased forms—catarrhal (laryngotracheitis), skin, conjunctival, febrile.

Considering the multi-linking of the pathogenetic chain of FESLF, the possibility of interrupting the infectious process at its various stages depending on the virulence of the pathogen, on the one hand, and the degree of resistance of the patient’s organism, on the other hand, determine the variety of clinical forms of this disease [38,39]. When the physiological barrier of the mucous membranes of the digestive tract is normal, the infection may interrupt at the earliest stage of its development and be limited to the clinical symptoms of gastrointestinal dysfunction. This is exactly what is observed with gastroenteritis and catarrhal forms, which can elude epidemiological registration. In other cases, in the absence of sufficiently pronounced changes in the entrance gates of the infection, the disease can immediately appear with general infection symptoms, which occurs with a febrile form of FESLF. In cases where the reactivity of the organism is high enough, the clinical picture is dominated by infectious and allergic manifestations—a rash and arthralgia without pronounced secondary focal changes (scarlet-like and arthralgic forms), with a fairly rapid development of post-infectious immunity and recovery of the patient. However, most often FESLF proceeds with the development of a typical symptom complex of the disease when both general infection, allergic symptoms and secondary focal lesions of various organs and systems are pronounced. In such cases, in severe and moderate courses of FESLF, a combined form of infection is usually diagnosed, for which relapses and exacerbations of the disease are especially characteristic.

A distinctive sign of the FESLF pathomorphology is the abscess-like or necrotic foci [40,41], which are found mainly in the lymph nodes, liver, spleen, and lungs, and have the appearance of yellowish or grayish-white tubercles with a diameter of 0, 1 to 1 cm or more containing a curd-like mass.

Granulomatous inflammation in FESLF is the most typical tissue reaction, similar in humans and animals. A.P. Avtsyn et al. [42] paid attention to the characteristic tendency of pseudotuberculous tubercles to necrosis and purulent fusion and called them granulomas with central karyorrhexis. Due to the latter, a large amount of nuclear detritus forms in the center of the granuloma. We examined the morphogenesis of pseudotuberculous granuloma in the experimental material upon infection with a virulent *Y. pseudotuberculosis* 3260 strain of serotype Ib isolated from a patient with FESLF and containing two plasmids, pYV with a molecular weight of 48 MDa and pVM82 (LD_50_ was 5 × I0^4^ m.c./mouse) [39,43]. Using the fluorescent antibody method, the value of the damaging effect of the FESLF causative agent during hematogenous dissemination in the organism as a trigger mechanism for the initiation of granulomatous inflammation was proved [43]. According to the cellular composition, pseudotuberculous granuloma was mixed. It was dominated by lymphocytes and macrophages, but there were also epithelioid cells, neutrophils and eosinophils, sometimes single giant multinuclear cells of the Pirogov-Langhans type. Granulomas were often formed against the background of tissue infiltration by polymorphic cells, mainly mononuclear cells and eosinophils. In the late stages of the infection (21–30 days post infection), a fibrous capsule formed around the granuloma, clearly delineating it from the surrounding tissue.

A previously unknown mechanism was revealed for the interaction of the FESLF causative agent with neutrophilic granulocytes, called the non-phagocytic type of resistance [44].

Its essence lies in the fact that recovery from *Y. pseudotuberculosis* infection is facilitated not by a phagocytic reaction, easily suppressed by the pathogen, but by the death of neutrophilic granulocytes as a result of karyorhexis with the accumulation in the inflammation foci of the decay products of their nuclei. The killing of the *Y. pseudotuberculosis* pathogen, provided by an arginine-rich fraction of the nuclear histone of neutrophilic granulocytes, has an antimicrobial effect.

Thus, the clinical-morphological nature of the disease allows us to attribute FESLF to the group of granulomatous diseases. The morphogenesis of acute granulomatous inflammation reflects the sequential participation of immediate and delayed hypersensitivity reactions, which are realized with the involvement of different populations of innate immunity cells. A number of signs have been identified that indicate the role of allergy in the pathogenesis of granulomatous inflammation in FESLF [43]. Mononuclear cells predominate in mature granulomas, mediating delayed-type hypersensitivity reactions, which is consistent with data [45] on the determining value of cellular immunity in the development of resistance to *Y. pseudotuberculosis.* The tendency of granulomas to peripheral growth by fusion with the newly formed granulomas is characteristic of FESLF, which is probably due to tissue sensitization as a result of repeated influx of specific antigens into them in the phase of repeated bacteremia. In experimental infection, it was established [43] that in the mild course of the disease, granulomatous inflammation proceeds with a predominance of delayed-type hypersensitivity reactions and granuloma encapsulation, in which, according to [44], the *Y. pseudotuberculosis* killing occurs under the influence of decay products of leukocytes. In severe courses of the *Y. pseudotuberculosis* infection associated with septic complications, the exudative and necrotic changes characteristic of immediate hypersensitivity reactions are more pronounced [43]. The inflammatory process in FESLF is a bright illustration of the functioning of a unified immunophagocytic system, which provides the organism with the ability to overcome the defects of individual links of immune defense and to repair damaged tissues. 

When studying cellular damages caused by *Y. pseudotuberculosis*, data were obtained that supplemented the morphological picture of granulomatous inflammation in target organs [46]. It has been established that the so-called “necrotic granulomas with central karyorrhexis” characteristic of FESLF without a pronounced perifocal inflammation, delimited by a connective tissue capsule, is a result of focal damage to effector cells of inflammation proceeding both as necrosis and apoptosis. Most likely, the combination of cell apoptosis and necrosis in the dynamics of the infectious process creates a peculiarity of inflammatory changes in FESLF, originally described as “micro-abscesses,” or “abscess-like or necrotic foci” based on the detection of cellular detritus in them. With sufficient reason, it can be considered that karyorrhexis mainly due to cell apoptosis, and not necrosis, causes the absence of confluent inflammatory foci and true abscess formation [39]. The similar nature of cell damages in FESLF determines the generally successful outcome of this generalized infection with gradual eradication of the pathogen and relatively rapid repair of damaged tissues, which may explain the rarity of generalized inflammatory complications and minimal lethality in this disease (less than 0.1%), according to [42]. 

## 5. Plasmid-Associated Virulence of *Y. pseudotuberculosis* and the FESLF Infectious Process 

For humans, pathogenic *Y. pseudotuberculosis* has a wide range of pathogenicity factors determined by chromosomal and plasmid genes [1,47,48,49]. By introducing new data on genetic diversity within the *Yersinia pseudotuberculosis* complex, authors [50,51] have reported a monomorphic clade that caused FESLF in the USSR in 1959, which often undergoes recombination possibly due to the conjugative plasmid pVM82 and contains the TcpI virulence protein. This previously unknown virulence protein, responsible for the unique clinical syndrome of FESLF, has significant sequence homology to members of the Toll/IL-1 receptor family (TIR) [50]. 

When studying the plasmid profile of 791 *Y. pseudotuberculosis* strains of serovar 1, plasmids were detected in 784 (99.1%) strains, which were isolated in 1977–1985 from sick people, wild animals, and washes from products and equipment of vegetable stores in various regions of the USSR [52]. Among these plasmids, the most frequently detected were the virulence plasmid *Y. pseudotuberculosis* (pYV) with a molecular weight of 48 MDa, and a plasmid with a weight of 82 MDa (pVM82) specific for *Y. pseudotuberculosis* of I serovar, which is the most common etiological agent of FESLF in humans and is not found in other representatives of the genus *Yersinia.*

Similar results were obtained when studying the plasmid characteristics of 212 *Y. pseudotuberculosis* strains isolated from various sources in 2000–2010 [8]. In addition to the pYV virulence plasmid and pVM82 plasmid, *Y. pseudotuberculosis* strains isolated from patients contained the YPMa superantigen, regardless of the disease severity. Between 2000 and 2010, the total average number of newly FESLF registered cases was 6024 per year, and the average frequency was 4.2 per 100,000 of the population [8].

The presence of the PYV virulence plasmid is a prerequisite for the pathogenicity of *Yersinia* spp. In the presence or absence of this plasmid, the strains are divided into pathogenic (G1-G3, G5-G6) and non-pathogenic subgroups (G4) [7]. The most frequent clinical genotype in Russia and Japan is G3, which possesses PYV and produces the superantigenic YPMa toxin, but does not have an island of high pathogenicity HPI [9,53].

Most Far Eastern strains of *Y pseudotuberculosis* produce YPMa, but not all cases of *Y pseudotuberculosis* infection are accompanied by the development of the FESLF form, which suggests an interaction between superantigen and other virulence factors [7]. Genome sequencing of the strain causing FESLF showed, besides pYV, additional virulence factors that may be involved in the pathogenesis of the disease [54]. These genomic elements include the following: plasmid pVM82 and *Yersinia* adhesion pathogenicity island (YAPI), which encodes type IV pili [55].

As noted above, the presence of two plasmids in *Y. pseudotuberculosis* with a molecular weight of 82 and 45–48 MDa determines the special virulence of the strains and causes a moderate and severe course of FESLF [52]. These authors found that the presence of the only PYV plasmid most often determines the sporadic incidence. The prevalence of epidemic outbreaks can be caused by both dominant plasmid types—pYV and pYV: pVM82, contained in the genome of the main etiological agent of pseudotuberculosis in people in Siberia and the Far East and *Y. pseudotuberculosis* of 1 serovar [10]. Moreover, the single-plasmid type of *Y. pseudotuberculosis*, containing only plasmid pYV, is characterized by outbreaks with a more favorable course, the prevalence of mild and erased forms of the disease. The presence of the additional plasmid pVM82 in strains of *Y. pseudotuberculosis* (plasmid type pYV: pVM82) leads to a more severe clinical course of the disease with multiple organ pathology [10,52]. In addition, it was shown that among natural strains of *Y. pseudotuberculossis*, plasmid p57 with a molecular weight of 57 MDa was found, which does not correlate with the epidemicity of the strains and is a fragment of plasmid pVM82 together with a DNA fragment of 25 MDa. It was found [56] that, in the presence of plasmid pVM82, the formation of antibodies to a number of the main antigens of *Y. pseudotuberculossis* is suppressed, while strains containing plasmid p57 do not have this effect. There are reports of the plasmid pVM82 effect on the clinical and morphological manifestations of FESLF [57,58].

To study the plasmid-associated virulence of the FESLF causatine agent, we reproduced an experimental infection caused by various plasmid types of *Y. pseudotuberculosis* [59]. For intraperitoneal infection of non-inbred white mice weighing 18–20 g, strains of four plasmid types *Y. pseudotuberculosis*, obtained from the collection of the Somov Research Institute of Epidemiology and Microbiology, were used: (1) strain H-5015 T^+^ (82^+^:48^+^) containing two plasmids pVM82 and pYV, LD_50_ was 3.7 × 10^7^ m.c./mouse; (2) its isogenic strain H-5015 T^-^ (82^+^:48^−^) containing a single plasmid pVM82; (3) strain H-5013 T^+^ (82^−^: 48^+^), containing only the plasmid pYV, LD_50_ was I, 4 × 10^8^ m.c./mouse; (4) its isogenic plasmid-free strain H-5013 T^−^ (48^−^). 

The obtained results showed the dependence of the histopathology of pseudotuberculosis infection on the plasmid type of pathogens [59]. The highest mortality rate (80%) was observed during experimental infection with the strain H-5013 T^+^ (plasmid type 48^+^: 82^−^) *Y. pseudotuberculosis*, carrying only the virulence plasmid pYV. It was characterized by inflammatory changes in organs with a predominance of mononuclear cells, starting from 1-day post-infection, and the development from three days of pronounced delymphatization of the spleen and lymph nodes, indicating a pronounced immunosuppression. In cases of infection caused by the *Y. pseudotuberculosis* strain H-5015^+^ (plasmid type 82^+^: 48^+^), the mortality rate was two times lower compared with the infection with the one-plasmid strain 5013 T^+^ (48^+^), when inflammatory changes were observed involving neutrophils and macrophages. Similar mortality rates were obtained in oral infection in rabbits caused by the same plasmid types *Y. pseudotuberculosis*, carrying one plasmid pYV and two plasmids pYV: pVM82 [60]. When infected with the *Y. pseudotuberculosis* strain H-5015 T^−^ (plasmid type 82^+^: 48^−^), carrying only the plasmid pVM82, the pathology in the organs was manifested by less acute inflammation with a predominance of mononuclear cells and the formation of granulomas, without hypoplasia of lymphoid organs, with minimal necrotic tissue changes [59].

In addition, in an experimental infection caused by different plasmid types of *Y. pseudotuberculosis*, the functional state of innate immunity cells was evaluated by the activity of lactate dehydrogenase (LDH), an oxygen-dependent system enzyme, using the Loyd method in own modification [61], and the level of nitric oxide (NO) metabolites [62]. By the activity of ATPase, the plasmalemma exoenzyme, it was found that only *Y. pseudotuberculosis* strains containing plasmid pVM82 exerted a stimulating effect on peritoneal exudate cells (neutrophils and macrophages), especially in combination with the virulence plasmid pYV (plasmid types 82^+^: 48^+^ and 82^+^: 48^−^) [62]. A more pronounced increase in the activity of LDH and especially the level of NO metabolites occurred in response to infection with the plasmid type pYV^−^: pVM82^+^ compared with the type pYV^+^: pVM82^+^ containing the additional plasmid virulence pYV on seven days post-infection. LDG and NO were 56.12 ± 0.002 and 80.98 ± 0.03 in response to infection with pYV^−^: pVM82^+^, whereas with infection with pYV^+^: pVM82^+^ was 8.02 ± 0.003 and 20.39 ± 0.01, respectively. At the same time, the plasmid-free *Y. pseudotuberculosis* strain (pYV^−^: pVM82^−^) had an activating effect on the production of lactate dehydrogenase (94.1 ± 0.04) and also caused an increase in the level of nitric oxide metabolites (66.5 ± 0.04) [62]. It was concluded that the presence of plasmid pVM82 in the FESLF causative agent provides effective immune defense by activating oxygen-dependent and notably nitroxide-dependent bactericidal mechanisms of innate immunity cells.

*Y. pseudotuberculosis* is known to cause apoptosis in macrophage culture [63]. In vivo, after intraperitoneal infection of out-bred white mice with the *Y. pseudotuberculosis* virulent strain 3260 (plasmid type pYV^+^: pVM82^+^) isolated from a patient with FESLF, we revealed the apoptosis-inducing effect of bacteria on blood leukocytes and peritoneal exudate cells, as well as on macrophages in granulomas in target organs (lungs, liver, spleen, and lymph nodes) [46]. In response to infections with a strain containing a single plasmid pVM82 (plasmid type pYV^−^: pVM82^+^), apoptosis in the cells of inflammatory foci prevailed, which was consistent with the above-noted high LDH activity marking apoptosis [64]. The *Y. pseudotuberculosis* virulent strain, containing two plasmids (type pYV^+^: pVM82^+^) mainly caused necrosis of phagocytes.

Thus, the pathogenetic significance of the plasmid pVM82 *Y. pseudotuberculosis* is associated with: the predominant stimulation of nitroxide-dependent bactericidal activity of innate immunity cells, the limitation of the generalized inflammatory response and the induction of granulomatous inflammation, and a decrease in the severity of pathogen-associated damage to lymphoid organs. 

## 6. Toxin Formation as the Most Important Factor in the Pathogenicity of the FESLF Pathogen

The biological characteristics of pathogens are factors that determine the specifics of pathological changes in infectious diseases, and among these factors the toxin formation is of great importance [65]. Based on the complexity of the biological properties of *Y. pseudotuberculosis*, it was concluded that the FESLF pathogen produces several secreted toxins and is associated with the bacterial cell, the expression of which contributes to the initiation of the disease [21]. The combined effect of pathogenicity factors with toxic function enhances the invasiveness of *Y. pseudotuberculosis* and ensures the passage of bacteria through the epithelium into the plate of the intestinal wall, and then through the vascular endothelium into the blood and lymph. It was found that high invasiveness is closely related to the enzymatic activity and toxigenicity of *Y. pseudotuberculosis*. By producing hyaluronidase, neuraminidase, and hemolysins [66,67], *Yersiniae* can cause depolymerization of the main intercellular substance to overcome mucous barriers, penetrating into the cells and vascular system of the organism. Components of the *Y. pseudotuberculosis* cell wall—lipolysaccharide having the properties of endotoxin, as well as a pore-forming protein—porin isolated from cytoplasmic membrane, possess toxic properties [68,69].

By the beginning of the 2000s, an idea was formed of the spectrum of *Y. pseudotuberculosis* toxins and some mechanisms for the realization of the toxic effect of the pathogen were discovered [70,71]. Two toxins of *Y. pseudotuberculosis*, thermolabile lethal toxin (TLT) and thermostable lethal toxin (TCT) are essential in the pathogenesis of FESLF.

The *Y. pseudotuberculosis* thermolabile toxin, a species protein with a molecular weight of 200 kDa, possessing immunogenic and allergenic properties, is capable of causing a local dermonecrotic reaction and death in laboratory animals upon parenteral introduction [71]. TLT was isolated by N.F. Timchenko from the *Y. pseudotuberculosis* strain 2517 of the third serovar containing plasmid pYV (a strain obtained from H. Mollaret, France), as well as from a plasmid-free strain. The dose-dependent selective effect of this toxin on the functional activity of innate immunity cells was established [72]. The activation of oxygen-dependent enzymes of mice neutrophils (lactate dehydrogenase and myeloperoxidase) was detected in the early period of contact (up to 3 h). In turn, there was an increase in the number of apoptotic macrophages in the early stages of contact with TLT (5 h) and their high level during the observation period (21 days) with a simultaneous increase in cytochrome oxidase activity and nitric oxide production [72]. Pathomorphological studies have shown [73] that this toxin is directly related to the development of infectious-toxic shock in *Y. pseudotuberculosis* infection.

The *Y. pseudotuberculosis* thermostable lethal toxin, a protein with a molecular weight of 45 kDa, was produced by 82.6% *Y. pseudotuberculosis* strains of I-VI serovars isolated from patients with FESLF and from the environment, at a culture temperature of 6–10 °C and 37 °C [71]. Moreover, a greater output of this protein was observed at low temperatures. Toxin production was observed in bacteria carrying both the pYV virulence plasmid and in combination with the pVM82 plasmid, as well as in plasmid-free bacteria, which suggests the chromosomal determination of TcT in the FESLF causative agent [71]. The toxin exerted a concentration-dependent effect on the activity of antioxidant enzymes (catalase, glutathione reductase), apoptosis and the viability of rat blood neutrophils, the apoptosis developed against the background of increased activity of these enzymes [74]. In in vivo and in vitro models, it was found that TcT *Y. pseudotuberculosis* changes the intensity of lipid peroxidation [75], inhibits protein synthesis in eukaryotic cells, reduces the phagocytic activity of neutrophils and macrophages, and has a depressing effect on antibody formation [71,76]. 

Out-bred mice weighing 15–20 g after intraperitoneal introduction of TcT *Y. pseudotuberculosis* at a dose of 20 μg developed toxinemia with the appearance of general tremor, convulsions, diarrhea, prostration and death of animals within 15–22 hours from the start of the experiment [71]. A morphological study showed that this toxin causes a pronounced multi-organ pathology with a predominance of degenerative-destructive changes. An assumption was made [71] that the formation of typical granulomas was due to the direct damaging effect of TcT on target organs. Ultrastructural studies have confirmed the membranolytic effect of this toxin on the cells of the parenchymal organs. Finally, it was recently established [77,78] that some isolates of *Y. pseudotuberculosis* produce a cytotoxic necrotizing factor (CNFY), but the functional consequences of this toxin on host-pathogen interactions during infection are little known. The presence of the cytotoxic necrotizing factor gene (*cnfY*) was detected in all 104 studied *Y. pseudotuberculosis* strains isolated in the Russian Federation in 1966–2015 from patients with FESLF, from rodents, and from environmental objects [79].

When studying the biological activity of CNFY on eukaryotic Hep-2 cells, it was found [79] that the toxin caused replication of the cell nucleus without its division. As a result, large multinucleate cells appeared in the culture, as shown previously [80]. For the first time, data were obtained on the biological effect of CNFY toxin on eukaryotic cells of the Vero E6 line with the formation of pronounced filopodia and lamellopodia, which indicates the activation of GTP proteins Cdc42 and Rac and can lead to apoptosis of cells [79].

The CNFY toxin is thermoregulated and is highly expressed in all colonized lymphatic tissues and organs in orally infected mice [78]. In the CNF-deprived variant obtained from the natural toxin-expressing *Y. pseudotuberculosis* isolate, the ability to spread to the mesenteric lymph nodes, liver and spleen was severely compromised, and lethality was completely lost. The CNFY toxin significantly contributed to the induction of acute inflammatory reactions and the formation of necrotic sites in infected tissues [78]. The presence of CNFY led to a strong reduction of professional phagocytes and natural killer cells, in particular in the spleen, while the loss of toxins caused effective tissue infiltration by immune cells and created bacteria that were completely avirulent, which led to their clearance from the host organs [81]. These data indicate the role of the CNFY toxin in the pathogenicity of the FESLF causative agent, suppression of the host (patient) antibacterial response and increased severity of this infection [78]. CNFY was predominantly expressed at 37 °C in a nutrient-rich medium resembling conditions found in the intestinal tract of mammals, including humans. 

Of great importance was the establishment of the role of the CNFY toxin in the transition of *Y. pseudotuberculosis* to persistence by reducing the inflammation induced by it [81,82]. Suppression of CNFY function increased interferon-γ-mediated responses, including non-inflammatory antimicrobial activity, which was accompanied by premature reprogramming of the pathogen’s transcriptional response to persistence [82].

Thus, the Russian strains of *Y. pseudotuberculosis*, the causative agent of FESLF as well as the European strains, contain the *cnfY* gene which determines the production of the cytotoxic necrotizing factor. It was noted [79] that the production of CNFY can lead to apoptosis of the host cells and the inability of the pathogen to use its reserve for its own life and reproduction. This indirectly suggests the ability of the pathogen to persist, given the presence of etiopathogenetic prerequisites to the development of persistent *Y. pseudotuberculosis* infection [83,84]. 

## 7. Conclusions

During the second half of the 20th century, manifestations of pseudotuberculosis (FESLF) were noted in the form of large epidemic outbreaks in the Far East of Russia [11], and in the 1960s this region determined almost the entire incidence of pseudotuberculosis infection in the former USSR. In the XXI century, according to the data of the reference center for monitoring pseudotuberculosis and yersiniosis of Pasteur Research Institute of Epidemiologe and Microbiology (St. Petersburg), yersinioses—pseudotuberculosis and intestinal yersiniosis—remain relevant for healthcare in Russia and many foreign countries [85,86]. Yersinioses are recorded in most constituent entities of the Russian Federation, however, the nosoareal of diseases is characterized by the presence of territories in the Far East, Siberia, the Ural, and the North West of the country that consistently have the highest incidence rate [86]. In 2010–2015 the highest incidence rates, exceeding the average Russian level by 2–4.7 times, were characteristic of the Far Eastern Federal District, the Siberian Federal District and the North-West Federal District, including St. Petersburg. In these federal districts, the range of pseudotuberculosis incidence rates ranged from 1.43% to 7.53% ooo. 

For the period from the beginning of official registration to the present (1992–2015), long-term incidence rates of pseudotuberculosis have significantly decreased from 8.3 in 1992 to 0.7% in 2015, and since 2013 there has been a trend towards its stabilization at a low level with an average incidence rate of 0.82% ± 0.05% ooo [86]. The reduction in the incidence rate is due to a reduction in the number of children in organized collectives involved in outbreaks due to the timely implementation of a set of preventive and anti-epidemic measures. On the other hand, social and economic transformations in society are having an effect. Currently, the epidemic process of pseudotuberculosis is characterized by a decrease in the proportion of outbreaks and the predominance of sporadic cases [86]. Pseudotuberculosis affects children to a greater extent. The proportion of registered cases of the disease among the children’s population is 62.8–77.5%, which is 1.7–3.4 times the proportion of sick adults.

Currently, *Y. pseudotuberculosis/Y. pestis* are considered as a unique, genetically identical pair, which provides an opportunity for in-depth study and comparative analysis of pathological processes associated with a high degree of pathogenicity of yersinioses causative agents. Far Eastern scarlet-like fever, prevalent mainly in Russia and Japan, began to attract worldwide attention as an emergent infection with unpredictable consequences. The discovery in 1959 in Vladivostok of FESLF, the causative agent of which is associated with a specific clonal line of *Y. pseudotuberculosis*, laid the foundation for new developments in a promising scientific field which concerns the mechanisms of the existence of pathogenic bacteria in various living conditions. These developments primarily include the phenomenon of *Y. pseudotuberculosis* psychrophiliety, which is a key property of the FESLF pathogen, as well as the sapronoses causative agents in general [19]. Based on the *Yersinia pseudotuberculosis* and *Listeria monocytogenes* models, studies of the academician G.P. Somov Science school paved the way for the development of the doctrine of sapronoses (saprozoonoses), the causative agents of which have a dual nature, saprophytic and parasitic properties, providing them with the opportunity to inhabit both the human organism and warm-blooded animals, and be present in the environment. Only the continuous circulation of bacteria between them ensures the existence of such pathogens in nature as a biological species [23,24].

The priority achievements of Russian researchers in the study of FESLF also include the discovery of the genetic and biochemical mechanisms of *Y. pseudotuberculosis* adaptation to changing environmental conditions, as well as the ultrastructural foundations of pathogen heteromorphism under these conditions. The fundamental importance is the evidence that the clinical-epidemic manifestation of pseudotuberculosis in the form of FESLF occurred as a result of the acquisition by *Y. pseudotuberculosis* of special extrachromosomal genetic information—a plasmid with a molecular weight of 82 MDa (plasmid pVM82) which determines the high epidemicity of the pathogen strains [52,56]. The pathogenetic significance of plasmid pVM82 is reflected in the morphogenesis of the infection process of FESLF, as well as in the effectiveness of cellular responses of its innate immune defense. Recently, the relevance of further research on the problem of FESLF has been associated with the study of dormant forms of *Y. pseudotuberculosis* and the idea of pseudotuberculosis as a persistent infection.

## References

[1] Galindo C.L., Rosenzweig J.A., Kirtley M.L., Chopra A.K. (2011). Pathogenesis of *Y. enterocolitica* and *Y. pseudotuberculosis* in human yersiniosis. J. Pathog..

[2] Knapp W. (1968). Die Pseudotuberkulose des menschen. Ther. Umsch..

[3] Mollaret H.H. (1965). Le laboratirie dahs le diagnostic d’infection humaine a bacilli de Malasser et Vignal. Gaset. Med. Paris.

[4] Zganjer M., Roic G., Cizmic A., Pajic A. (2005). Infectious ileocecitis—Appendicitis mimicking syndrome. Bratisl Lek List..

[5] Tertti R., Vuento R., Mikkola P., Granfors K., Makela A., Toivanen A. (1989). Clinical manifestation of *Yersinia pseudotuberculosis* in children. Eur. J. Clin. Microbiol. Infect. Dis..

[6] Grant H., Rode H., Cywes S. (1994). *Yersinia pseudotuberculosis* affecting the appendix. J. Pediatr. Surg..

[7] Amphlett A. (2015). Far East Scarlet-like Fever: A review of the epidemiology, symptomatology and role of superantigenic toxin: *Yersinia pseudotuberculosis*-derived mitogen. Open Forum Infect. Dis..

[8] Tseneva G.Y., Chesnokova M.V., Voskresenskaya E.A., Klimov V.T., Kokorina G.I., Burgasova O.A., Sayapina L.V., Tirskikh K.A., Karimova T.V. (2012). The Y. pseudotuberculosis infections in the Russian Federation. Adv. Exp. Med. Biol..

[9] Fukushima H., Matsuda Y., Seki R. (2001). Geographical heterogeneity between Far East and Western countries in prevalence of the virulence plasmid, the superantigen *Yersinia pseudotuberculosis* derived mitogen and the high-pathogenicity island among *Yersinia pseudotuberculosis* strains. J. Clin. Microbiol..

[10] Somov G.P., Pokrovsky V.I., Besednova N.N., Antonenko F.F. (2001). Pseudotuberculosis.

[11] Somov G.P. (1979). Far Eastern Scarlet-Like Fever.

[12] Korolyuk A.M. (2017). The scientific feat of the military microbiologist: To the 50th anniversary of the experience of self-infection with pseudotuberculosis. Bull. Ros. S.M. Kirov Mil. Med. Acad..

[13] Timchenko N.F., Ermolaeva S.A., Adgamov R.R., Kuznetsova N.A. The causative agent of Far Eastern Scarlet-like Fever (human pseudotuberculosis) is a clone of Yersinia poseudotuberculosis. Proceedings of the 3rd All-Russian Scientific-Practice Conference with International Participation “Infections due to Yersinia”.

[14] Timchenko N.F., Adgamov R.R., Popov A.F., Psareva E.K., Sobyanin K.A., Gintsburg A.L., Ermolaeva S.A. (2016). Far East Scarlet-Like Fever caused by a new related genotypes of *Yersinia pseudotuberculosis*, Russia. Emerg. Infect. Dis..

[15] Achtman M., Zurth K., Morelli C., Torrea G., Guiyoule A., Carniel E. (1999). *Yersinia pestis*, a cause of plaque, is a recently emerged clone of *Yersinia pseudotuberculosis*. Proc. Natl. Acad. Sci. USA.

[16] Carniel E. From *Y. pseudotuberculosis* to *Y. pestis*. Proceedings of the 3rd All-Russian Scientific-Practice Conference with International Participation “Infections due to Yersinia”.

[17] Somov G.P., Varvashevich T.N. (1984). Enzymatic mechanisms of the psychrophilicity of a pseudotuberculosis microbe. Zhurnal Mikrobio. Epidemiol. Immunobiol..

[18] Avtsyn A.P., Zhavoronkov A.A. (1978). Some issues of infectious pathology in the BAM zone. Clin. Med..

[19] Somov G.P., Varvashevich T.N., Timchenko N.F. (1991). Psychophilicity of Pathogenic Bacteria.

[20] Timchenko N.F., Antonenko F.F. (1990). The entry portals and routes of *Yersinia pseudotuberculosis* penetration into the warm-blooded organism. Zhurnal Mikrobiol. Epidemiol. Immunobiol..

[21] Timchenko N.F., Venediktov V.S., Pavlova T.N. (1988). Simulation of the initiation of pseudotuberculosis infection. Zhurnal Mikrobiol. Epidemiol. Immunobiol..

[22] Timchenko N.F., Somov G.P. (1986). Pathogenetic significance of the psychrophilicity of Y. pseudotuberculosis. Zhurnal Mikrobiol. Epidemiol. Immunobiol..

[23] Somov G.P. (2001). Modern understanding of sapronoses (main results of the study of the problem). Pac. Med. J..

[24] Somov G.P., Litvin V.Y. (1988). Saprophytism and Parasitism of Pathogenic Bacteria (Ecological Aspects).

[25] Somov G.P., Buzoleva L.S. (2004). Adaptation of Pathogenic Bacteria to Abiotic Environmental Factors.

[26] Buzoleva L.S., Chumak A.D. (2000). Use of poly-β-hydroxybutyric acid by the bacteria *Yersinia pseudotuberculosis* and Listeria monicytogenes at different temperatures. Microbiology.

[27] Buzoleva L.S., Krivosheeva A.M., Isachenko A.S., Somova L.M., Somov G.P. (2006). Effect of temperature on synthesis of polyphosphates in *Yersinia pseudotuberculosis* and Listeria monocytogenes under starvation conditions. Biochemistry.

[28] Tafelshtein E.E., Golubinsky E.P., Maramovich A.S., Popov A.V. (1995). Adaptive mechanisms in the ecology of pseudotuberculosis microbe. Med. Parasitol. Parasit. Dis..

[29] Somov G.P. (1997). Ecological features of extraorganismal populations of pathogenic bacteria. Zhurnal Mikrobiol. Epidemiol. Immunobiol..

[30] Somova L.M., Buzoleva L.S., Plekhova N.G. (2009). Ultrastructure of Pathogenic Bacteria in Different Ecological Conditions.

[31] Isachenko A.S., Isachkova L.M., Buzoleva L.S., Pustovalov E.V., Somov G.P. (2000). Ultrastructure of *Yersinia pseudotuberculosis* bacteria with prolonged dwelling in soil. Bull. Exper. Biol. Med..

[32] Somova L.M., Buzoleva L.S., Isachenko A.S., Somov G.P. (2006). Adaptive ultrastructural changes in bacteria *Yersinia pseudotuberculosis* when living in soil. Zhurnal Mikrobiol. Epidemiol. Immunobiol..

[33] Dorman C.J. (1996). Flexible response: DNA supercoiling, transcription and bacterial adaptation to environmental stress. Trends Microbiol..

[34] Wang J.Y., Syvanen M. (1992). DNA twist as a transcriptional sensor for environmental changes. Mol. Microbiol..

[35] Watson S.P., Clements M.O., Foster S.J. (1998). Characterization of the starvation survival response of Staphilococcus aureus. J. Bacteriol..

[36] Volova T.G., Kalacheva G.S., Puzyr A.P. (1996). The effect of the intracellular pool of polyoxybutyrate on the growth of hydrogen bacteria under non-optimal conditions. Microbiology.

[37] Gabdrakhmanova L.A., Balaban N.P., Sharipova M.R., Tokmakova Y.S., Sokolova E.A., Rudenskaya G.N., Leshchinskaya I.B. (2002). Optimization of cultivation medium for the production of Bacillus intermedius 3-19 glutamyl endopeptidase. Microbiology.

[38] Avtsyn A.P., Isachkova L.M., Zhavoronkov A.A., Somov G.P., Makhnev M.V. (1990). The main features of the pathogenesis of pseudotuberculosis. Arch. Pathol..

[39] Somova L.M., Antonenko F.F. (2019). Pseudotuberculosis (Clinical-Morphological Aspects).

[40] Anichkov N.M. (1972). Morphogenesis of experimental pseudotuberculosis infection. Cand. Med. Sci. Diss. Leningrad..

[41] Avtsyn A.P., Zhavoronkov A.A. (1980). Pathology of Yersinioses. Arch. Pathol..

[42] Avtsyn A.P., Zhavoronkov A.A., Marachev A.G., Milovanov A.P. (1985). Human Pathology in the North.

[43] Isachkova L.M. (1990). Pseudotuberculosis (patho- and morphogenesis). Doct. Med. Sci. Diss. Moscow.

[44] Mazing Y.A. (1982). Histological and cytochemical characteristics of experimental and spontaneous pseudotuberculosis infections. Cand. Med. Sci. Diss. Leningr..

[45] Besednova N.N. (1979). Experimental and clinical-immunological study of pseudotuberculosis infection. Doct. Med. Sci. Diss. Moscow.

[46] Somova L.M., Plekhova N.G., Drobot E.I. (2012). New aspects of the pathology of pseudotuberculosis. Arch. Pathol..

[47] Tseneva G.Y., Solodovnikova N.Y., Voskresenskaya E.A. (2002). Molecular Aspects of Yersinia Virulence. Clin. Microbiol. Antimicrobial. Chemother..

[48] Voskresenskaya E.A., Tseneva G.Y., Kozarenko A.A., Ivanishenko A.P., Litvinova L.V., Shimanskaya E.L. (2009). Genetic characteristics of *Yersinia pseudotuberculosis* and the epidemiological features of pseudotuberculosis group diseases in organized groups. Epidemiol. Infect. Dis..

[49] Francis M.S. (2013). The pathogenic *Yersiniae*—Advances in the understanding of physiology and virulence. Front. Cell. Infect. Microbiol..

[50] Norenberg D., Wieser A., Magistro G., Hoffmann C., Meyer C., Messerer M., Schubert S. (2013). Molecular analysis of a novel Toll/interleukin-1 receptor (TIR)-doman containing virulence protein of *Y. pseudotuberculosis* among Far East scarlet-like fever serotype I strains. Intern. J. Med. Microbiol..

[51] Savina S., Martin L., Bouchier C., Filali S., Chenau J., Zhou Z., Becher F., Fukushima H., Thomson N.R., Scholz H.C. (2014). The *Yersinia pseudotuberculosis* complex: Characterization and delineation of a new species, *Yersinia wautersii*. Int. J. Med. Microbiol..

[52] Shubin F.N., Sibirtsev Y.T., Rasskazov V.A. (1985). Plasmids *Yersinia pseudotuberculosis* and their significance in the implementation of the epidemic process in pseudotuberculosis. J. Microbiol. Epidemiol. Immunobiol..

[53] Carnoy C., Mullet C., Muller-Alouf H., Emmanuelle L. (2000). Superantigem YPMa exacerbates the virulence of *Yersinia pseudotuberculosis* in mice. Infect Immun..

[54] Eppinger M., Rosovitz M.J., Fricke W.F., Rasko D.A., Kokorina G., Fayolle C., Lindler L.E., Carniel E., Ravel J. (2007). The complete genome sequence of *Yersinia pseudotuberculosis* IP31758, the causative agent of Far East scarlet-like fever. PLoS Genet..

[55] Collyn F., Lety M.A., Nair S., Escuyer V., Ben Y.A., Simonet M., Marceau M. (2002). Yersinia pseudotuberculosis harbors a type IV pilus gene cluster that contributes to pathogenicity. Infect. Immun..

[56] Gunzburg A.L., Shubin F.N., Shovadaeva G.A., Kulichenko A.N., Yanishevsky N.V. (1988). A new trait of pathogenicity encoded by plasmid pVM 82 *Yersinia pseudotuberculosis*. Genetics.

[57] Shurygina I.A., Malov A.V., Maramovich A.S., Klimov V.T. (2001). The effect of a plasmid with a molecular weight of 82 MDa on the clinical and morphological manifestations of pseudotuberculosis. Sib. Med. J..

[58] Shurygina I.A., Chesnokova M.V., Klimov V.T., Malov I.V., Maramovich A.S. (2003). Pseudotuberculosis.

[59] Somova L.M., Shubin F.N., Plekhova N.G., Drobot E.I., Lyapun I.N. (2019). Inflammation induced by different plasmid types of russian *Yersinia pseudotuberculosis* strains. Russ. J. Infect. Immun..

[60] Isachkova L.M., Zhavoronkov A.A., Antonenko F.F. (1994). Pathology of Pseudotuberculosis.

[61] Loyda Z., Gossrau R., Schiebler T. (1982). Histochemistry of Enzymes, Laboratory Methods.

[62] Somova L.M., Lyapun I.N., Drobot E.I., Shubin F.N., Plekhova N.G. (2018). The Metabolic Activity of Innate Immunity Cells in Experimental Infection Caused by Various Plasmid Types of *Yersinia pseudotuberculosis*. Biochem. Mol. Biol..

[63] Monack D.M., Mecsas J., Ghori N., Fackow S. (1997). Yersinia signals macrophages to undergo apoptosid and YopJ is necessary for cell death. Proc. Natl. Acad. Sci. USA.

[64] Rydell-Tormanen K., Ulter L., Mahanty S., Sachez A.J., Wagoner K.D. (2006). Direct evidence of secondary necrosis of neutrophils during intense lung. Eur. Respir..

[65] Ezepchuk Y.V. (1985). Pathogenicity as a Function of Biomolecules.

[66] Varvashevich T.N., Sidorova V.E., Potapova I.Y. (1983). Enzymes neuraminidase and hyaluronidase as a model for studying the mechanisms of adaptation of Yersinia. Yersiniosis.

[67] Timchenko N.F. (1972). Materials on the microbiological characteristics of the causative agent of Far Eastern scarlet-like fever (human pseudotuberculosis). Cand. Med. Sci. Diss Vladivostok.

[68] Kuznetsova T.A., Kovalevskaya A.M., Sanina O.P., Besednova N.N. (1985). Effect of lipopolysaccharide of the pseudotuberculosis bacterium on the functional activity of macrophages. Antibiot. Med. Biotechnol..

[69] Sidorova V.E., Timchenko N.F. (1974). The study of the toxic properties of surface and extracellular proteins of a pseudotuberculosis microbe. Far Eastern Scarlet Fever-Like Fever.

[70] Timchenko N.F. (2011). The development of ideas about the pathogenicity factors of the causative agent of Far Eastern scarlet-like fever (human pseudotuberculosis). Bull. SB RAMS.

[71] Timchenko N.F., Nedashkovskayam E.P., Dolmatova L.S., Somova-Isachkova L.M. (2004). Toxins Yersinia Pseudotuberculosis.

[72] Plekhova N.G., Drobot E.I., Timchenko N.F., Somova L.M., Persiyanova E.N. (2014). Effect of thermolabile toxin from *Yersinia pseudotuberculosis* on functions of innate immunity cells. Bull. Exp. Biol. Med..

[73] Somova L.M., Plekhova N.G., Drobot E.I., Nedashkovskaya E.P., Timchenko N.F. (2010). Pathomorphological changes in experimental toxinemia caused by the thermolabile toxin *Yersinia pseudotuberculosis*. Pac. Med. J..

[74] Dolmatova L.S., Zaika O.A., Nedashkovskaya E.P., Timchenko N.F. (2010). Investigation of the mechanisms of the apoptosis-modulating effect of the thermostable toxin *Yersinia pseudotuberculosis* and the corrective effect of the extract from Far Eastern holothuria species on rat neutrophils in vitro. Pac. Med. J..

[75] Raznik S.D. (1999). Characterization of the biological effect of the thermostable toxin *Yersinia pseudotuberculosis*. Cand. Med. Sci. Diss. Vladivostok.

[76] Logvinenko A.A. (2000). The effect of the thermostable toxin *Yersinia pseudotuberculosis* on the immune system (experimental study). Cand. Med. Sci. Diss. Vladivostok.

[77] Persiyanova E.V., Adgamov R.R., Surin A.K., Psareva E.K., Ermolaeva S.A. (2013). The cytotoxic necrotizing factor *Yersinia pseudotuberculosis*, the causative agent of Far East Scarlatiniform fever. Bull. SB RAMS..

[78] Schweer J., Kulkarni D., Kochut A., Pezoldt J., Pisano F., Pils M.C., Genth H., Huehn J., Dersch P. (2013). The cytotoxic necrotizing factor of *Yersinia pseudotuberculosis* (CNFY) enhances inflammation and Yop delivery during infection by activation of Rho GTPases. PLoS Pathog..

[79] Psareva E.K., Timchenko N.F., Ermolaeva S.A. (2017). Cytotoxic necrotizing factor *Yersinia pseudotuberculosis* as an agent of pseudotuberculosis infection. Infect. Dis..

[80] Lockman H.A., Gillespie R.A., Baker B.D., Shakhnovich E. (2002). *Yersinia pseudotuberculosis* produces a cytotoxic necrotizing factor. Infect. Immun..

[81] Heine W., Beckstette M., Heroven K., Thiemann S., Heise U., Nuss A.M., Pisano F., Strowig T., Dersch P. (2018). Loss of CNF_Y_ toxin-induced inflammation drives *Yersinia pseudotuberculosis* into persistency. PLoS Pathog..

[82] Wolters M., Boyle E.C., Lardong K., Trulzsch K., Steffen A., Rottner K., Ruckdeschel K., Aepfelbacher M. (2013). Cytotoxic necrotizing factor-Y boosts Yersinia effector translocation by activating Rac protein. J. Biol. Chem..

[83] Somova L.M., Andryukov B.G., Timchenko N.F., Psareva E.K. (2019). Pseudotuberculosis as a persistent infection: Etiopathogenetic prerequisites. J. Microbiol. Epidemiol. Immunobiol..

[84] Fahlgren A., Avican K., Westermark L., Nordfelth R., Fällman M. (2014). Colonization of cecum is important for development of persistent infection by *Yersinia pseudotuberculosis*. Infect. Immun..

[85] Zhebrun A.B., Yersinioses in the Russian Federation (2014). News Bulletin.

[86] Totolyan A.A., Yersinioses in the Russian Federation (2017). News Bulletin.

